# BCL9 and C9orf5 Are Associated with Negative Symptoms in Schizophrenia: Meta-Analysis of Two Genome-Wide Association Studies

**DOI:** 10.1371/journal.pone.0051674

**Published:** 2013-01-29

**Authors:** Chun Xu, Nagesh Aragam, Xia Li, Erika Cynthia Villla, Liang Wang, David Briones, Leonora Petty, Yolanda Posada, Tania Bedard Arana, Grace Cruz, ChunXiang Mao, Cynthia Camarillo, Brenda Bin Su, Michael A. Escamilla, KeSheng Wang

**Affiliations:** 1 Texas Tech University Health Sciences Center, Paul L. Foster School of Medicine, Departments of Psychiatry/Neurology and the Center of Excellence in Neuroscience, El Paso, Texas, United States of America; 2 Department of Biostatistics and Epidemiology, College of Public Health, East Tennessee State University, Johnson City, Tennesee, United States of America; 3 Department of Microbiology and Immunology, Division of Infectious Diseases, School of Medicine, University of North Carolina at Chapel Hill, Chapel Hill, North Carolina, United States of America; 4 College of Bioinformatics Science and Technology, Harbin Medical University, Harbin, People’s Republic of China; 5 University of Texas at El Paso, El Paso, Texas, United States of America; 6 Walden University, Minneapolis, Minnesota, United States of America; 7 University of Toronto, Toronto, Canada; Leibniz-Institute for Arteriosclerosis Research at the University Muenster, Germany

## Abstract

Schizophrenia is a chronic and debilitating psychiatric condition affecting slightly more than 1% of the population worldwide and it is a multifactorial disorder with a high degree of heritability (80%) based on family and twin studies. Increasing lines of evidence suggest intermediate phenotypes/endophenotypes are more associated with causes of the disease and are less genetically complex than the broader disease spectrum. Negative symptoms in schizophrenia are attractive intermediate phenotypes based on their clinical and treatment response features. Therefore, our objective was to identify genetic variants underlying the negative symptoms of schizophrenia by analyzing two genome-wide association (GWA) data sets consisting of a total of 1,774 European-American patients and 2,726 controls. Logistic regression analysis of negative symptoms as a binary trait (adjusted for age and sex) was performed using PLINK. For meta-analysis of two datasets, the fixed-effect model in PLINK was applied. Through meta-analysis we identified 25 single nucleotide polymorphisms (SNPs) associated with negative symptoms with p<5×10^−5^. Especially we detected five SNPs in the first two genes/loci strongly associated with negative symptoms of schizophrenia (P_meta-analysis_<6.22×10^−6^), which included three SNPs in the BCL9 gene: rs583583 showed the strongest association at a P_meta-analysis_ of 6.00×10^−7^ and two SNPs in the C9orf5 (the top SNP is rs643410 with a p = 1.29 ×10^−6^). Through meta-analysis, we identified several additional negative symptoms associated genes (*ST3GAL1, RNF144, CTNNA3* and *ZNF385D*). This is the first report of the common variants influencing negative symptoms of schizophrenia. These results provide direct evidence of using of negative symptoms as an intermediate phenotype to dissect the complex genetics of schizophrenia. However, additional studies are warranted to examine the underlying mechanisms of these disease-associated SNPs in these genes.

## Introduction

Schizophrenia (SCZ) is one of the most tragic psychiatric disorders with a high degree of genetic and clinical heterogeneity and a prevalence of approximately 1% worldwide. Given its debilitation, morbidity, mortality and economic burden, SCZ represents a major public health concern and an important topic in health research. Schizophrenia is known to be a multifactorial disorder with a demonstrated heritability of 80% in family studies and meta-analysis of multiple twin studies [1], [2]. While the number of genes potentially implicated in SCZ has been estimated to be over 1000, the progress in the identification of susceptibility genes has been slow. Previous functional studies have not been successfully replicated, which may be due, at least in part, to the inherent limitations associated with assessing phenotypic heterogeneity, using too broad phenotypes, or a lack of consideration of the effect of the disease-associated single nucleotide polymorphisms (SNPs) on gene regulation. This highlights an urgent need for re-thinking strategic, complementary approaches to identify true biomarkers for the disorder. Molecular genetics technology development and the results from the recent genome-wide association (GWA) meta-analyses suggest a number of genes/loci to be associated with the broader SCZ spectrum of phenotypes; however, with regard to the identified causative variants, it is likely that genetic variants with a more definitive effect on SCZ phenotypes are still missing.

It has been well documented that using intermediate phenotypes may aid in the genetic dissection of neuropsychiatric diseases like SCZ (see review in [3]). Intermediate phenotypes, in contrast to broad phenotypes, appear to follow simple inheritance patterns allowing for genetic linkage and association study of patients [4]. Therefore, using an intermediate phenotype approach permits mapping of the effect of individual risk genes based on neurobiological parameters, known to be associated with causes of SCZ rather than targeting the effects of the disorder, which are believed to be less genetically complex than those associated with the disorder itself [4]. A recent study with a focus on a number of SCZ-related intermediate phenotypes (e.g., neurophysiological and neurocognitive data), examined 1536 SNPs and identified a number of genetic variants that were associated with 12 SCZ intermediate phenotypes. This finding supports the use of relevant intermediate phenotypes and the bootstrap total significance test for identifying genetic variation underlying the etiology of SCZ [5], and provides evidence that using SCZ-related intermediate phenotypes will increase the chance of detecting genetic linkage and association not feasible with the broader spectrum of SCZ [3], [4]. Among the SCZ intermediate phenotypes, negative symptoms of SCZ are of particular interest. One feature of the negative symptoms of SCZ is its high degree of specificity and sensitivity, which is responsible for major impairments in functionality, and often has an onset before other diagnostic criteria are present [6]. Moreover, negative symptoms are the most potent predictor for a poor long-term outcome [7]. The therapeutic response of negative symptoms to antipsychotic treatment has been reported as being mild to almost absent [8]. Among patients with negative symptoms, each may present with a different combination of symptoms and likewise, have a distinctive evolution and response to treatment.

SCZ genetic association studies suggest an association between negative symptoms of SCZ and the Cys311 allele in *DRD2* gene, and this may account for the observed differences in negative symptoms presentation (see review in [9]). Furthermore, results of a pharmacogenetics study suggested an association between the variants of type-three metabotropic glutamate receptor gene (*GRM3*) and negative symptoms of SCZ improvement when the patients were treated with clozapine [10]. The authors concluded that the *GRM3* genetic variants may be useful predictors of negative symptom improvement in persons treated with olanzapine as well. Another pharmacogenetic study also demonstrated that patients with a Ser311Cys genotype of DRD2 gene showed significant improvements with a larger change in scores for negative symptoms of SCZ as compared to patients with a Ser311Ser genotype over the course of 42 days of treatment with risperidone [11]. Further, an animal study using the D2R-OE mouse model [12] and the clinical observations in human subjects [13], [14] demonstrate that the severity of cognitive symptoms correlated more highly with the negative symptoms than with the positive symptoms of SCZ. Moreover, a recent study using a genetic imaging approach demonstrated epistatic interactions between the FGF mouse and the glypican 1 gene on brain development. The authors concluded that this model may be useful for investigating the negative symptoms of SCZ [15].

Based on the genetics, pharmacological and imaging studies discussed above, we hypothesize that there is a genetic basis for the presence of negative symptoms among patients with SCZ. The conventional GWA study approach is a powerful tool to identify disease-related genes for many common human disorders and other phenotypes (Wellcome Trust Case Control Consortium 2007 [16]). Recently, GWA experiments identified several genes associated with SCZ (such as *ZNF804A*, *NRGN*, *RELN*, *TCF4*, and variants in the *MHC*) and yielded remarkable new experimental evidence leading to a better understanding of the genetic models and the biological risk factors involved in SCZ [2], [17]. Until now, no study has focused on GWA analysis of negative symptoms of SCZ. In the present study we use two existing GWA-data sets (729,454 markers) and large sample sizes (1774 cases with negative symptoms of SCZ and 2726 controls) to identify novel variants associated with negative symptom-susceptibility.

## Results

### Genotype Quality Control and Descriptive Statistics

We removed SNPs with HWE p<0.00001 or call rates <95% or minor allele frequency (MAF) <1%. In total, 722,112 SNPs were left for the GAIN sample and 711,137 SNPs were remained for the nonGAIN sample. The details about these subjects were described elsewhere [18]–[20]. After merging SNP genotype data with phenotype data and removing outlier individuals based on the principal-component analysis, 950 cases with negative symptoms and 1,378 controls (1,298 males and 1,040 females) were left in the GAIN sample while 824 cases and 1,348 controls (1,258 males and 924 females) were left in the nonGAIN sample (Table 1).

**Table 1:** Descriptive Characteristics of Negative Symptoms of SCZ and Controls.

	Negative Symptoms		Controls	
	GAIN	nonGAIN	GAIN	nonGAIN
Number	950	824	1378	1348
Sex, N (%)				
Males	664 (69.9%)	588 (70.1%)	634 (46%)	670 (49.7%)
Females	296 (30.1%)	246 (29.9%)	744 (54%)	678 (50.3%)
Age, years				
Mean ± SD	43.8±11.2	43.0±11.7	51.1±16.9	49.8±15.8
Range	15–86	18–79	18–90	18–90

SCZ, schizophrenia.

### Genome-wide Association Analysis of Negative Symptoms of SCZ

We identified 25 SNPs associated with negative symptoms at p values <5×10^−5^ in the meta-analysis (Table 2). These 25 SNPs were located with and/or near 18 different genes/loci. The best disease-associated SNP rs583583 (p = 6.0×10^−7^, Table 2) at 1q21.2 within B-cell CLL/lymphoma 9 (*BCL9*) gene. Interestingly, two SNPs (rs583583 and rs828836) in this gene were found to be located at biologically conserved regions among the different species according to the UCSC Genome browser (http://genome.ucsc.edu/, NCBI36/hg18). The second interesting locus was rs643410 (p = 1.29×10^−6^, Table 2) at 9q31 within *C9orf5*. One (rs838836) of three SNPs on this locus was located at a conserved region. The *C9orf5* gene has been shown to be associated with prostate cancer [21]. The third disease-associated gene identified was the ST3 beta-galactoside alpha-2,3-sialyltransferase 1 (*ST3GAL1*, also called *SIAT4A*) and it was also associated with negative symptoms of SCZ (p = 1.75×10^−5^, Table 2).

**Table 2:** Top 25 SNPs Associated with Negative Symptoms in the Meta-Analysis.

CHR	SNP	Position	Gene	Location	Allele^a^	Conserved	P_meta^b^	Q^c^	MAF^d^	OR^e^	P__GAIN_ f	EMP2^g^	MAF^h^	OR^i^	P_nonGAIN_ j	EMP2^k^
1	rs583583	145549738	BCL9	1q21.2	A	Conserved	6.00×10^−7^	0.670	0.28	1.30	7.67×10^−5^	0.0049	0.30	1.297	2.06×10^−3^	0.048
9	rs643410	110839384	C9orf5	9q31	A	Nonconserved	1.29×10^−6^	0.776	0.05	1.60	2.08×10^−4^	0.00699	0.05	1.51	1.75×10^−3^	0.041
9	rs838836	110794109	C9orf5	9q31	A	Conserved	1.32×10^−6^	0.915	0.05	1.55	6.08×10^−4^	0.011	0.05	1.58	6.42×10^−4^	0.012
8	rs2860223	134737859	Near ST3Gal1	8q24.22	C	Nonconserved	2.46×10^−6^	0.926	0.35	1.26	3.66×10^−4^	0.008	0.38	0.8	2.06×10^−3^	0.048
1	rs2275552	145598569	BCL9	1q21.2	T	Nonconserved	6.08×10^−6^	0.665	0.28	1.22	2.43×10^−3^	0.038	0.29	1.28	7.1×10^−4^	0.013
1	rs6674938	145598089	BCL9	1q21.2	A	Conserved	6.22×10^−6^	0.695	0.28	1.23	2.20×10^−3^	0.032	0.29	1.27	8.01×10^−4^	0.015
3	rs6599101	40422552	ENTPD3	3p	A	Conserved	1.35×10^−5^	0.456	0.02	0.30	4.13×10^−4^	0.008	0.02	0.43	8.05×10^−4^	0.153
8	rs11786962	27895165	SCARA5	8p21.1	T	Conserved	1.46×10^−5^	0.287	0.27	1.31	9.55×10^−5^	0.006	0.28	1.18	3.0×10^−2^	0.467
10	rs16924239	68646147	CTNNA3	10q22.2	C	Conserved	1.64×10^−5^	0.725	0.03	1.82	3.19×10^−4^	0.0069	0.04	0.60	1.65×10^−2^	0.295
8	rs2945741	134715811	Near ST3GA	8q24.22	A	Nonconserved	1.75×10^−5^	0.031	0.26	1.37	3.69×10^−6^	0.0009	0.28	0.91	1.88×10^−1^	0.980
7	rs7808050	144592154	Near LOC643308	7q35	A	Conserved	2.16×10^−5^	0.045	0.44	1.12	7.27×10^−2^	0.770	0.41	1.35	1.41×10^−5^	0.0009
8	rs2616157	20728567	Near RNU3P2	8p21.3	T	Conserved	2.17×10^−5^	0.768	0.19	0.76	9.54×10^−4^	0.015	0.19	0.79	7.27×10^−3^	0.139
16	rs9932309	5605457	Near NPM1P3	16p13.3	C	Conserved	2.33×10^−5^	0.527	0.28	0.78	4.08×10^−4^	0.008	0.24	1.20	1.60×10^−2^	0.286
14	rs17112308	26699127	Near RPS27AP4	14q12	A	Conserved	2.35×10^−5^	0.496	0.10	0.67	5.77×10^−4^	0.011	0.08	1.34	1.09×10^−2^	0.207
12	rs10880017	74665362	Near NAP1L1	12q21.2	T	Conserved	2.75×10^−5^	0.995	0.47	0.83	1.97×10^−3^	0.028	0.46	0.83	4.62×10^−3^	0.099
17	rs4985969	20890280	Near USP22	17p11.2	C	Conserved	3.04×10^−5^	0.317	0.25	1.18	1.95×10^−2^	0.308	0.25	1.30	3.14×10^−4^	0.005
3	rs7624777	106716829	ALCAM	3q13.1	C	Nonconserved	3.12×10^−5^	0.440	0.29	1.19	1.17×10^−2^	0.198	0.28	1.28	6.77×10^−4^	0.013
3	rs1585383	67939031	Near FAM19A1	3p14.1	T	Nonconserved	3.14×10^−5^	0.895	0.21	1.23	3.29×10^−3^	0.057	0.22	1.25	3.19×10^−3^	0.073
8	rs10103263	20729747	Near RNU3P2	8p21.3	C	Conserved	3.29×10^−5^	0.821	0.19	0.77	1.42×10^−3^	0.018	0.19	0.79	7.66×10^−3^	0.145
9	rs10119049	111406928	Near PALM2	9q31.3	T	Conserved	3.51×10^−5^	0.620	0.32	1.24	8.17×10^−4^	0.015	0.33	1.19	1.31×10^−2^	0.242
3	rs2060531	21492494	ZNF385D	3p24.3	A	Conserved	3.72×10^−5^	0.112	0.42	0.88	4.74×10^−2^	0.604	0.43	0.76	7.80×10^−5^	0.003
3	rs9869330	21492680	ZNF385D	3p24.3	T	Nonconserved	4.02×10^−5^	0.105	0.42	0.88	5.01×10^−2^	0.627	0.43	0.76	7.62×10^−5^	0.003
2	rs10167072	6990386	RNF144	2p25.2	G	Nonconserved	4.06×10^−5^	0.547	0.28	1.19	9.24×10^−3^	0.151	0.29	1.26	1.21×10^−3^	0.023
7	rs6957015	131634470	PLXNA4	7q32,3	A	Conserved	4.16×10^−5^	0.250	0.12	1.41	1.74×10^−4^	0.0069	0.13	1.21	4.41×10^−2^	0.623
9	rs2583377	110908598	C9orf5	9q31	A	Nonconserved	4.22×10^−5^	0.723	0.05	1.51	1.28×10^−3^	0.016	0.05	1.42	1.06×10^−2^	0.202

a Minor allele;

b p-value for the meta-analysis;

c p-value for Cochrane’s Q statistic;

d Minor allele frequency in the GAIN sample;

e Odds ratio for the GAIN sample;

f p-value for the GAIN sample based on logistic regression;

g corrected empirical p-value for the GAIN sample generated by 100,000 permutation tests using Max (T) permutation procedure implemented in PLINK;

h Minor allele frequency in the nonGAIN sample;

i Odds ratio for the nonGAIN sample;

j p-value for the nonGAIN sample based on logistic regression;

k corrected empirical p-value for the nonGAIN sample generated by 100,000 permutation tests using Max (T) permutation procedure implemented in PLINK.

However, only one SNP (rs10167072) in the *RNF144* gene was associated with negative symptoms of SCZ (p = 4.06×10^−5^, Table 2). One previous study reported that a SNP (rs6741819) in the *RNF144* gene mediated the metabolic side effects of the antipsychotic drug (risperidone) at genome wide significant level (p = 2.43×10^−7^) in 738 patients with SCZ [22].

One intronic SNP (rs16924239) at the *CTNNA3* gene was located at a conserved genomic region and was shown to be significantly associated with negative symptoms of SCZ (p = 1.64×10^−5^, Table 2).

The other two associated SNPs (rs2060531 and rs9869330) lie within an interesting candidate gene and may warrant further investigation: the negative symptoms-associated gene was *ZNF385D*, which is located at 3p24. Two SNPs from this gene showed significant associations with negative symptoms of SCZ with p values of 3.72×10^−5^ and 4.02×10^−5^ respectively.

**Figure 1:**
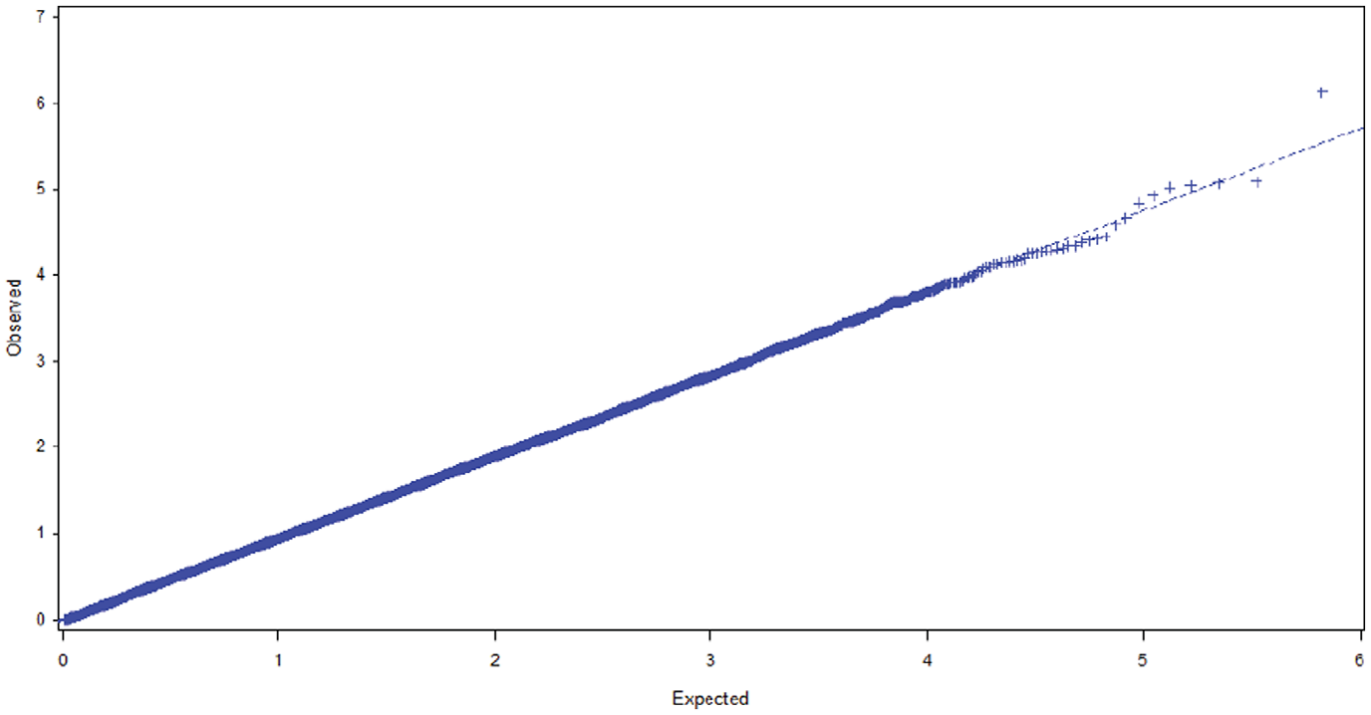
Quantile-quantile (Q-Q) plot of the observed versus expected –log _10_ (p) values for the meta-analysis. Y-value: observed test statistic and X-value: expected statistic under the global null hypothesis of no association.

All p-values based on Q statistics were larger than 0.05 (except for rs2945741 with p = 0.031), which indicated that there was no heterogeneity for these SNPs between the GAIN and the nonGAIN samples (Table 2). The Q-Q plot for meta-analyses is presented in Figure 1. Table 2 also revealed that most SNPs were associated with negative symptoms of SCZ in both the GAIN and the nonGAIN samples (p<0.05). Applying a permutation procedure for multiple test correction also yielded significant p values (Table 2, corrected empirical p-values). Eighteen of the top 25 SNPs for the GAIN sample and 12 of the top 25 SNPs for the nonGAIN sample had corrected p<0.05 after permutation tests (Table 2).

Next, we thought it might be interesting to test disease-associated variants between the SCZ with and without negative symptoms, however, sample size is small for the SCZ without negative symptoms (183 and 242 individuals for GAIN and nonGAIN samples, respectively), clearly, it will limit power to adequately test for association.

### In silico Analysis

After observing that the negative symptoms-associated SNPs were located in intron regions of the candidate genes/loci, we evaluated whether these variants were located in regions of the gene that might have potential functional importance. The sequences containing the associated SNPs were examined for microRNA binding sites, splicing sites, regulatory gene regions, and species-conserved regions. We hypothesized that the negative symptoms-associated common variants in species-conserved regions may be more functionally important than common variants in non-conserved regions. Using the UCSC Genome browser (http://genome.ucsc.edu/, NCBI36/hg18), we found that fifteen out of 25 disease-associated SNPs were located at species-conserved regions (Table 2).

Furthermore, we performed gene ontology analysis to see whether any gene ontology categories are overrepresented among negative symptoms of SCZ associated-SNPs with low p values [23]. Results of gene ontology analysis for negative symptoms of SCZ associated SNPs with p _Meta-analysis_<3.04×10^−5^, gene ontology categories with enrichment p-values <0.05 are shown in Table 3.

**Table 3:** Results of Gene Ontology Analysis for Negative Symptoms of SCZ Associated SNPs with P_Meta-analysis_<3.04×10^−5^, Gene Ontology Categories with Enrichment P-values <0.05.

Pathway-ID	Pathway Name	P-value	Genes
path:00603	Glycosphingolipid biosynthesis - globo series	0.0109	ST3GAL1
path:00533	Glycosaminoglycan biosynthesis - keratan sulfate	0.0116	ST3GAL1
path:00604	Glycosphingolipid biosynthesis - ganglio series	0.0116	ST3GAL1
path:00512	O-Glycan biosynthesis	0.0232	ST3GAL1
path:05213	Endometrial cancer	0.0398	CTNNA3

## Discussion

Given the animal study [15], clinical [7], pharmacological [8], [10], [11], and imaging genetic and psychiatric genetic studies [9], [24], we hypothesized that genetic variants will be differentially associated with negative symptoms of SCZ by examining the two existing GWA -SCZ data sets. As far as we know, this is the first report of a GWA study looking at negative symptoms in SCZ. We have identified several new genes associated with negative symptoms of SCZ. These major findings, for the first time, provide pilot evidence that supports the use of negative symptoms as an intermediate phenotype to dissect the complex genetics of schizophrenia. It is possible that multiple genes may contribute to the negative symptoms of SCZ susceptibility. However, additional studies are warranted to examine the underlying mechanisms of these disease-associated genes.

Although none of the SNPs reached the genome-wide significance threshold (p<5×10^−8^), many SNPs were very close to this level. Three SNPs, rs583583, rs2275552 and rs6674938, in the BCL9 gene at chromosome 1q21.1 showed significant associations to the negative symptoms of SCZ, with rs583583 being the strongest of all markers tested in the meta-analyses. It is noteworthy that the *BCL9* gene is expressed in all tissues and has two transcripts: a major 6.3-kb transcript and a less prominent 4.2-kb transcript [25]. One previous study reported that the BCL9 protein is required for efficient T-cell factor–mediated transcription in the Wnt signaling pathway [26], which has been suggested to be involved in the pathophysiology of mental disorders using animal studies [27]. Furthermore, increasing lines of evidence suggests that the Wnt signaling pathway influences neuroplasticity, cell survival, and adult neurogenesis [27]. Recent human genetic studies also reported that some *BCL9* variants are associated with SCZ in the Asian population [28], but not associated with bipolar disorder (BD) in the Caucasian population [29]. Copy number variant (CNV) analysis also revealed that large recurrent microdeletions at 1q21.1 (where the BCL9 gene is located) were associated with SCZ [30]. Soon after, results showing the association of 1q21 in SCZ was replicated in 1,433 SCZ cases and 33,250 matched healthy controls in the Phase I study and 3,285 cases and 7,951 controls in a Phase II study [24], [31]. There is a well-documented, peer-reviewed body of scientific publications showing that CNVs in human chromosome 1q21, where the *BCL9* gene is located were strongly associated with SCZ (for review please see [32]) as well as rare CNVs (deletions and duplications) observed in SCZ in a number of independent studies (for review, please see [33]). It might be interesting to examine CNV on 1q21 among the patients with negative symptoms of SCZ in the future.

The second top signal came from the variants of *C9orf5* gene (also known as CG2; CG-2; *TMEM245*; *FLJ23668*; and *FLJ33224*) at 9q31. A previous genome-wide scan demonstrated linkage for an 11 cM region of human chromosome 9q31–33 for familial dysautonomia which is a disorder of the autonomic nervous system affecting the development and survival of sensory, sympathetic and some parasympathetic neurons in the autonomic and sensory nervous system [34]. In 1999, a novel human transcript CG-2 (*C9ORF5*), was isolated from the familial dysautonomia candidate region on 9q31 using a combination of cDNA selection while sequence analysis of CG-2 indicates that it is likely to encode a transmembrane protein and proposed to be a candidate for familial dysautonomia [35]. One recent study reported that miR-32, which maps to intron 14 of *C9orf5*, is found at significantly higher levels in prostate cancer tissue when compared to non-tumor prostate [21]. More recently, SNP rs523340 within the *C9orf5* was reported to be associated with information processing speed, which is an important cognitive function that is compromised in psychiatric illness (e.g., schizophrenia, depression) and old age; it shares genetic background with complex cognition (e.g., working memory, reasoning) [36].

The third signal in the current study, the negative symptoms-associated genetic variants of the *ST3GAL1* gene were also reported to be risk variants for bipolar disorder (BP) in a number of previous reports [37]–[39]. This is compelling because another ST3-related gene, *ST8SIA2*, which codes for one of the nerve cell adhesion molecules, and appears to play a key role in cell-cell interaction in the developing brain [40] was described as having an association with SCZ risk [41]. Moreover, according to our gene ontology analysis, ST3GAL1 contains several closely related GO categories/KEGG pathways, including O-Glycan biosynthesis and glycosphingolipid biosynthesis in our gene ontology analysis (Table 3). The latter has also been reported in SCZ according to one gene expression study, the authors concluded that the changing pattern of gene expression in glycosphingolipid biosynthesis could represent an adaptive response to the pathology of SCZ progression or is a compensatory effect to antipsychotic medication [42].

Of note, the *RNF144* variants showed association not only with negative symptoms in our current study, but also with metabolic side effects of antipsychotic drugs [22], therefore, it might be interesting to examine whether negative symptoms of SCZ carrying *RNF144* variants have different antipsychotic treatment response as compared with the patients without these variants in the future.

Another disease-associated gene is *CTNNA3*, which has been shown to be associated with other psychiatric disorders, such as general cognitive ability [43], and Alzheimer’s disease [44], [45]. *CTNNA3* is a key protein of the adherens junctional complex and plays a crucial role in cellular adherence [46]. Since this gene is located within a common fragile site, epigenetically regulated and transcribed through multiple promoters, it might be interesting to examine if epigenetic regulation is involved in the pathophysiology of negative symptoms in the future. Increasing lines of evidence indicate that the *CTNNA3* is also involved in neuronal migration, synaptogenesis and the formation of neuronal circuits [47]. In addition, slight migrational disturbances are often seen in the mesofrontal cortical brain structures of patients with epilepsy [48]. A recent review discussed that deficits in working memory in SCZ are attributable to specific pathological alterations in the neuronal circuitry of the dorsolateral prefrontal cortex [49]. In the future, function analysis and brain structure studies of negative symptoms of SCZ may provide a deeper understanding of the pathogenesis of this intermediate phenotype, particularly in patients who carry risk variants of the *CTNNA3*.

Two additional negative symptoms-associated SNPs (rs2060531 and rs9869330) at chromosome 3p24.3 were located in the *ZNF385D* gene, which also showed an association with BP in a previous report [50].

The remaining disease-associated genes/loci are less well understood in psychiatric disorders, like *FAM19A1, PLXNA4, LOC643308, RNU3P2, PALM2, SCARA5, RPS27AP4*, and *NPM1P3*. The *NAP1L1* and *USP22* variants, for example, are cancer-related genes/loci and the gene, *ALCAM* (also called *CD166*) was originally identified as a transmembrane receptor that is involved in T-cell activation, development, inflammation and transendothelial migration of neutrophils, and a cancer susceptible gene [51]. Therefore, further replication studies are required to validate if these genes/loci are involved in the development of negative symptoms of SCZ.

There are a number of strengths in this study. Our sample size is relatively large for this type of study and is relatively ethnically homogeneous. The current results of negative symptoms-associated SNPs together with the results in the in silico analysis (including gene ontology) provide insight into how multiple genes might contribute to disease biology of negative symptoms of SCZ. In addition, by applying more stringent criteria including the application of permutation analyses, we ensure that the findings are not due to any sample size imbalance or by chance. We also realized some limitations in our study: 1) none of the disease-associated SNPs reached genome-wide significance level and therefore, the current findings might be spurious or the results of a type I error despite the more stringent meta-analysis approach adopted in our analyses. Future confirmation studies using an independent sample and/or family-based association studies will provide an opportunity to more accurately dissect the genetic complexity of these genetic variants for negative symptoms of SCZ. It is important to note that a family study design is immune to population stratification, 2) there is a lack of heritability of negative symptoms in SCZ, therefore, future studies assessing the inheritability for this intermediate phenotype will help to dissect the complex genetics of schizophrenia, and 3) other variants, such as copy number variation (CNV, recent discoveries of putatively-causal structural abnormalities) and rare variants (for example, SNPs less than 1% of minor allele frequency) may not be captured using common SNPs. Therefore, future CNV analysis, next generation sequencing technologies (such as target gene sequencing on these 18 genes/loci) will provide an opportunity for in-depth molecular profiling of fundamental biological processes of the negative symptoms-associated variants in these potential genes/loci identified in the current study.

Taken together, our findings suggest a possible common genetic cause of negative symptoms in SCZ, and our current meta-analysis together with future replication studies in a more homogeneous and narrowly-defined group of patients will be able to reveal the genetic basis for negative symptoms of SCZ.

### Conclusion

This is the first report identifying common genetic variations associated with negative symptoms of schizophrenia using meta-analysis. The current study provides pilot evidence supporting the use of negative symptoms as an intermediate phenotype to dissect the complex genetics of schizophrenia. However, additional studies are warranted to examine the underlying mechanisms of disease-associated SNPs in these genes/loci.

## Materials and Methods

### Samples

Two GWAS data sets were used: 1) GAIN sample – 992 European-American (EA) patients with negative symptoms of SCZ and 1442 EA healthy controls were selected from the publicly available data from Genome-Wide Association Study of Schizophrenia - Study Accession: phs000021.v2.p1; 2) NonGAIN sample – 863 EA patients with negative symptoms of SCZ and 1364 EA controls were selected from Molecular Genetics of Schizophrenia - nonGAIN Sample (nonGAIN) - Study Accession: phs000167.v1.p1. NonGAIN sample is part of the Molecular Genetics of Schizophrenia (MGS) genome wide association study of 3,972 cases and 3,629 controls after quality control. Unrelated adult cases with DSM-IIIR (SGI study) or DSM-IV (MGS1, MGS2 studies) schizophrenia (SCZ) or schizoaffective disorder (SA) were collected under institutional review board-approved protocols in three studies, Schizophrenia Genetics Initiative (SGI), Molecular Genetics of Schizophrenia Part 1 (MGS1), and MGS23, as previously described in detail in [20] and phs000167.v1.p1.

Cases selected met criteria for SCZ or schizoaffective disorder per the Diagnostic and Statistical Manual of Mental Disorders version IV (DSM-IV). Negative symptoms of SCZ were defined as a binary trait (present or absent) for SCZ cases per DSM-IV. Schedule for Assessment of Negative Symptoms (SANS) was used as the clinical tool for the negative symptoms of SCZ as described in phs000167.v1.p1. Negative symptoms were defined as deficits of normal emotional responses or of other thought processes, and respond less well to medication. Negative symptoms commonly include flat or blunted affect and emotion, poverty of speech (alogia), inability to experience pleasure (anhedonia), lack of desire to form relationships (asociality), and lack of motivation (avolition). Research suggests that negative symptoms contribute more to poor quality of life, functional disability, and the burden on others than do positive symptoms [52].

Genotyping data using the Affymetrix Genome-wide human SNP Array 6.0 (total 729,454 SNPs) were available for both datasets.

### Statistical Methods

For the initial GWA analysis, HelixTree Software (http://www.goldenhelix.com/SNP_Variation/HelixTree/index.html, Golden Helix, Bozeman, MT) was used to assess control genotype data for conformity with Hardy-Weinberg equilibrium (HWE). To deal with population stratification, the principal-component analysis approach with ten principal components (Price et al., 2006) in HelixTree was used to identify outlier individuals. Then, logistic regression analysis of negative symptoms of SCZ as a binary trait, adjusted for age and sex, was performed for GAIN and nonGAIN samples using PLINK v1.07 [53]. The asymptotic p-value for this test was observed while the odds ratio with standard error was estimated. For logistic regression analyses, the additive model was applied. For statistical significance, we used a very conservative per test significance level of α = 5×10^−8^ (Wellcome Trust Case Control Consortium) [16]. We also used a less stringent criterion of “suggestive association” with a cut-off of α = 10^−5^. In addition to obtaining nominal *p*-values, empirical *p*-values were generated by 100,000 permutation tests using Max (T) permutation procedure implemented in PLINK. This procedure allows calculation of two sets of empirical significance values: pointwise estimates of an individual SNP’s significance (empirical pointwise *p*-values) and corrected values for multiple testing (corrected empirical *p*-values). Results from the two GWA analyses were directly meta-analyzed by combining the separate results of GAIN and nonGAIN samples (odds ratio and standard error of odds ratio) into one meta-analysis of overall effects. For meta-analysis of two datasets, the basic meta-analysis function in PLINK was applied. Fixed-effect meta-analysis p-value and fixed-effect OR were estimated.

### Statistical Power Analysis

With the combined sample size of both GWA studies, we calculated 97.1% of statistical power for negative symptoms of SCZ (N = 1774) and healthy controls (N = 2726) to detect an association of p = 5×10^−7^ based on the GWA significant threshold using Genetics Power Calculator [54] for a gene of moderate effect (OR = 1.75); however, the GWA significant threshold is too conservative since many SNPs are in strong linkage disequilibrium.

### Bioinformatics Analysis

We also examined whether these negative symptoms of SCZ-associated variants have an impact on gene function, including association with microRNA binding sites, being located at species-conserved regions and having functional importance in gene regulations using silico analysis: SNP Functional Portal (http://brainarray.mbni.med.umich.edu/Brainarray/Database/SearchSNP/snpfunc.aspx), SNP Function Prediction from NIEHS (http://snpinfo.niehs.nih.gov/snpfunc.htm) and miRdSNP (http://mirdsnp.ccr.buffalo.edu/search.php). The species-conserved regions for the SNPs were defined using the UCSC Genome browser (http://genome.ucsc.edu/, NCBI36/hg18).

Gene ontology analysis was also performed using the ALIGATOR method (Holmans et al., 2009) to investigate whether there was an enrichment for SNPs in genes in any gene ontology categories among the SNPs with low, but not genome-wide significant, p-values. We investigated these SNP sets using a threshold of P<3×10^−5^. The SNPs located within and/or close to genes/loci were included (based on NCBI SNP build 129 and NCBI sequence build 36.3). One SNP per gene, with the lowest p-value, was included in the ALIGATOR analysis using 20 000 simulated replicate gene lists and 5000 simulated replicate studies.
